# Functional coupling of sensorimotor and associative areas during a catching ball task: a qEEG coherence study

**DOI:** 10.1186/1755-7682-5-9

**Published:** 2012-02-24

**Authors:** Farmy Silva, Oscar Arias-Carrión, Silmar Teixeira, Bruna Velasques, Caroline Peressutti, Flávia Paes, Luis F Basile, Manuel Menéndez-González, Eric Murillo-Rodríguez, Mauricio Cagy, Roberto Piedade, Antonio Egídio Nardi, Sergio Machado, Pedro Ribeiro

**Affiliations:** 1 Brain Mapping and Sensory Motor Integration, Institute of Psychiatry of Federal University of Rio de Janeiro (IPUB/UFRJ), Rio de Janeiro, Brazil; 2 Division of Neurosurgery, University of São Paulo Medical School, São Paulo, Brazil; 3 Laboratory of Psychophysiology, Faculty of Psychology and Phonoaudiology - UMESP, São Paulo, Brazil; 4 Bioscience Department (EEFD/UFRJ), School of Physical Education, Rio de Janeiro, Brazil; 5 Division of Epidemiology and Biostatistics, Institute of Health Community, Federal Fluminense University (UFF), Rio de Janeiro, Brazil; 6 Institute of Applied Neuroscience (INA), Rio de Janeiro, Brazil; 7 Laboratory of Panic and Respiration, Institute of Psychiatry of Federal University of Rio de Janeiro (IPUB/UFRJ), Rio de Janeiro, Brazil; 8 National Institute of Translational Medicine (INCT-TM), Rio de Janeiro, Brazil; 9 Department for Translational Neurodegeneration, German Center for Neurodegenerative Diseases, Technical University Munich (TUM), Munich, Germany; 10 Faculty of Psychology, Brazilian Institute of Medicine and Rehabilitation (IBMR), Rio de Janeiro, Brazil; 11 Quiropraxia Program, Central University, Santiago, Chile; 12 Unit of Neurology, Hospital Álvarez-Buylla, Merida, Spain; 13 Laboratorio de Neurociencias Moleculares e Integrativas. Escuela de Medicina, División Ciencias de la Salud. Universidad Anáhuac Mayab. Mérida, Yucatán. México

## Abstract

**Background:**

Catching an object is a complex movement that involves not only programming but also effective motor coordination. Such behavior is related to the activation and recruitment of cortical regions that participates in the sensorimotor integration process. This study aimed to elucidate the cortical mechanisms involved in anticipatory actions when performing a task of catching an object in free fall.

**Methods:**

Quantitative electroencephalography (qEEG) was recorded using a 20-channel EEG system in 20 healthy right-handed participants performed the catching ball task. We used the EEG coherence analysis to investigate subdivisions of alpha (8-12 Hz) and beta (12-30 Hz) bands, which are related to cognitive processing and sensory-motor integration.

**Results:**

Notwithstanding, we found the main effects for the factor block; for alpha-1, coherence decreased from the first to sixth block, and the opposite effect occurred for alpha-2 and beta-2, with coherence increasing along the blocks.

**Conclusion:**

It was concluded that to perform successfully our task, which involved anticipatory processes (i.e. feedback mechanisms), subjects exhibited a great involvement of sensory-motor and associative areas, possibly due to organization of information to process visuospatial parameters and further catch the falling object.

## Introduction

In a dynamic environment, the Central Nervous System (CNS) is in constant activity because it receives external sensory stimuli all the time, in order to specifically maintain an appropriate motor performance [1]. Thus, in the CNS occurs an association between the ability to communicate with the external environment and encoding information for the internal control of the movement, aiming at an appropriate task execution [2]. These factors are elementary components for the preparation and adjustment of a motor act, besides they take part in the integration among different specialized centers in the final production of the movement [3].

Catching an object is a complex movement that involves not only programming but also effective motor coordination [4,5]. Such behavior is related to the activation and recruitment of cortical regions that participates in the sensorimotor integration process. Moreover, the CNS needs to capture information from the environment in order to prepare the motor act and to enhance the execution of goal-directed tasks, e.g., catching an object [3]. On the other hand, sensorimotor integration process occurs in the left hemisphere differently than in the right hemisphere. In particular, the right hemisphere is related to external environment, i.e., plays a dominant role on spatial functions, such as attention, perception, response selection, apart from information of limb position and posture [2,6]; while left hemisphere is associated with the internal environment, i.e., plays a dominant role on motor functions, such as motor planning [7] and the control of limb trajectory [6].

In other words, the functional specialization of cortical regions and the coupling between non specialized and specialized areas has been widely investigated [8-11]. The analysis of neural patterns associated with cognitive and sensorimotor processes has shown that the cooperation between cortical signals is a dynamic relationship [9]. Quantitative electroencephalography (qEEG) might be well suited to the task of monitoring changes in brain state that occur when an individual have to perform a sensorimotor task [12-14]. Furthermore, for understanding the mechanisms involved in sensorimotor integration and in the production of voluntary movements [1,11,15]. Based on this assumption, our experiment investigated the coherence of the qEEG in sensorimotor and associative areas in a catching task in which participants have to catch a ball in free fall. Coherence represents a measurement of linear covariation between two signals in the frequency domain [9].

Spectral features of the qEEG in the alpha (8-12 Hz) band are sensitive to variations in cognitive or sensorimotor actions [16,17]. With regard to the beta band, studies have shown an association with attention, movement control, processing information and sensorimotor integration [13,18,19]. Within this context, the assessment of differences in alpha (i.e., alpha 1 or slow-alpha [8-10 Hz] and alpha 2 or fast-alpha [10.1-12.5 Hz] and beta (i.e., beta 1 [13.0-19.5 Hz] and beta 2 [20.0-30.5 Hz]) sub-bands would be informative as it could address how the brain organizes and integrates sensory information, performs cognitive operations, and achieves motor control during the performance of a catching ball task. The study of alpha and beta sub-bands has shown qualitative differences.

Therefore, this study aims to bring new insights into the relationship between alpha and beta bands of qEEG within sensorimotor and associative areas involved in anticipatory actions, through coherence analysis, during a task in which individuals had to catch a free falling object (i.e., ball). In particular, through the subdivision of alpha (i.e., alpha 1 and alpha 2) and beta (i.e., beta 1 and beta 2), we tried to elucidate electrocortical mechanisms within four target regions, i) frontal (F3-F4, F3-FZ, F4-FZ); ii) central (C3-C4, C3-CZ, C4-CZ); iii) parietal (P3-P4, P3-PZ, P4-PZ); iv) and occipital (O1-O2, O1-OZ, O2-OZ). We hypothesized that due to the task features, e.g. manipulation of objects and stimuli identification, we will found different patterns of activation in alpha and beta bands during task execution.

## Methods

### Sample

Sample was composed of 20 healthy subjects (7 male and 13 female), right handed [20], with ages varying between 25 and 40 years old (mean: 32.5, SD: 7.5). Inclusion criteria were absence of mental or physical impairments, no history of psychoactive substances and no neuromuscular disorders (screened by a previous anamnesis and clinical examination). All subjects signed a consent form and were aware of the whole experimental protocol. The experiment was approved by the Ethics Committee of Federal University of Rio de Janeiro (IPUB/UFRJ).

### Task procedures

The task was performed in a sound and light-attenuated room, to minimize sensory interference. Individuals sat on a comfortable chair to minimize muscular artifacts, while electroencephalography and electromyography (EMG) data were collected. An electromagnetic system, composed of two solenoids, was placed right in front of the subject and released 8-cm balls, one at each 11 s, at 40 cm above the participant's hand, straight onto the subject's hand. The right hand was placed in a way that the four medial metacarpi were in the fall line. After its catch, the ball was immediately discharged. Each released ball composed a trial and blocks were made of 15 trials. All experiment had six blocks that lasted 2 min and 30 s with 1 min intervals between them.

### Data acquisition

Electroencephalography-The International 10/20 System for electrodes [21] was used with the 20-channel EEG system Braintech-3000 (EMSA-Medical Instruments, Brazil). The 20 electrodes were arranged in a nylon cap (ElectroCap Inc., Fairfax, VA, USA) yielding monopole derivations referred to linked earlobes. In addition, two 9-mm diameter electrodes were attached above and on the external corner of the right eye, in a bipolar electrode montage, for eye-movement (EOG) artifacts monitoring. Impedance of EEG and EOG electrodes were kept under 5-10KΩ. The data acquired had total amplitude of less than 100 μV. The EEG signal was amplified with a gain of 22,000, analogically filtered between 0.01 Hz (high-pass) and 100 Hz (low-pass), and sampled at 240 Hz. The software Data Acquisition (Delphi 5.0), developed at the Brain Mapping and Sensorimotor Integration Laboratory was employed to filter the raw data: notch (60 Hz), high-pass of 0.3 Hz and low-pass of 100 Hz.

Electromyography (EMG) activity of the flexor carpi radialis (FCR), flexor carpi ulnaris (FCU), extensor carpi radialis (ECR) and extensor carpi ulnaris (ECU) was recorded by an EMG device (Lynx-EMG1000), to monitor and assess any voluntary movement during the task. Bipolar electrodes (2 mm recording diameter) were attached to the skin. The reference electrode was fixed on the skin overlying the lateral epicondyle near the wrist joint. The skin was cleaned with alcohol prior to electrode attachment. The EMG was amplified (x1000), filtered (10-3000 Hz), digitized (10000 samples/s), and recorded synchronously to the EEG onto the computer's hard drive. In each trial, the EMG signal was rectified and averaged over 500 ms from the trigger point.

### Data processing

To quantify reference-free data, a visual inspection and independent component analysis (ICA) were applied to identify and remove any remaining artifacts, i.e., eye blinks and ocular movements produced by the task. Data from individual electrodes exhibiting loss of contact with the scalp or high impedances (> 10 kΩ) were deleted and data from single-trial epochs exhibiting excessive movement artifact (± 100 μV) were also deleted. Independent component analysis (ICA) was then applied to identify and remove any remaining artifacts after the initial visual inspection. ICA is an information maximization algorithm that derives spatial filters by blind source separation of the EEG signals into temporally independent and spatially fixed components. Independent components resembling eye-blink or muscle artifact were removed and the remaining components were then back-projected onto the scalp electrodes by multiplying the input data by the inverse matrix of the spatial filter coefficients derived from ICA using established procedures. The ICA-filtered data were then reinspected for residual artifacts using the same rejection criteria described above. Then, a classic estimator was applied for the power spectral density (PSD), or directly from the square modulus of the FT (Fourier Transform), which was performed by MATLAB (Matworks, Inc.). Quantitative EEG parameters were reduced to 4-s periods (the selected epoch started 2 s before and ended 2 s after the trigger, i.e., moment preceding balls drop and moment after balls drop). The electrophysiological measure analyzed was coherence. It represents a measurement of linear covariation between two signals in the frequency domain. It is mathematically bounded between zero and one, whereby one signifies a perfect linear association and zero denotes that the signals are not linearly related at that particular frequency. The premise is that when activities from spatially remote events covary they tend to interact, also denoted as functional connectivity. Standard coherence as a measure of functional coupling provides a link between two signals but no directional information. To this end, estimators can be constructed, such as a directed transfer function, which examines asymmetries in inter-regional information flow and establishes a direction of drive between the coupled sites [22,23].

### Frequency band and spatial electrode localization

The alpha band (8-12 Hz) is sensitive to variations in cognitive or sensorimotor actions [24,25]. The alpha band was divided in, alpha 1 or slow-alpha (8-10 Hz) and alpha 2 or fast-alpha (10-12 Hz). Slow-alpha is topographically widespread over the entire scalp, its functional meaning is less clear and it seems non-task specific; probably related to general task demands and attention processes such as alertness and expectancy; in contrast, fast-alpha (10-12 Hz) is topographically much more restricted and shows a clear relation to the cognitive task. Fast-alpha is related to sensory semantic encoding or long-term memory processes [26]. Moreover, beta band is associated with attention, movement control, processing information and sensorimotor integration [13,18,19]. The beta-band is divided in beta-1 (13.0 - 19.5 Hz), which are widespread lower frequency beta oscillations representing inhibitory components of cognitive control, and beta-2 (20.0 -30.5 Hz), which are higher frequency beta oscillations associated to response selection and activation, and are more specialized in terms of function and cortical distribution [27].

The F3, FZ and F4 electrodes represent the premotor cortex, functionally responsible for selection of movements, preparation and voluntary control of action [1,3]. The C3 and C4 electrodes are placed on the pre-central and central gyri, representing the primary sensory motor cortex (SM1) in each hemisphere that is functionally linked to motor preparation, perception and execution of movement [28]. The CZ electrode represents the SM1 of both hemispheres and the supplementary motor area (SMA), which is functionally related to temporal organization and coordination of sequential movements [29]. The P3, PZ and P4 electrodes represent the medial parietal areas, functionally related to integration of sensory information from different modalities and play important roles in the manipulation of objects, and in visuospatial processing (e.g., spatiotemporal coordination related to the contact hand-object) [15,30]. The O1, OZ and O2 electrodes represent the visual areas, functionally related to visual perception, stimuli identification and attention processes, mainly in dynamic environment involving objects detection which demands a high readiness state [31].

### Statistical analysis

The statistical design allowed for examination of functional connectivity and directionality of communication between sensorimotor areas in each hemisphere, with respective regions related to sensory, motor execution, and integrative or associative functions. All results are given as mean values and standard deviation. A two way ANOVA was used to analyze the within subject's factors moment (2 levels: preceding and after ball drop) and block (1 to 6) for each pair of electrodes: F3-F4, F3-FZ, F4-FZ, P3-P4, P3-PZ, P4-PZ, C3-C4, C3-CZ, C4-CZ, O1-O2, O1-OZ, O2-OZ (p < 0.05). Further analysis was implemented to observe F3-FZ, F4-FZ, P3-PZ, P4-PZ, C3-CZ, C4-CZ, O1-OZ, O2-OZ we use paired-test to analyze the difference between hemispheres within each block. For statistical analysis SPSS package was used.

## Results

**Figure 1:**
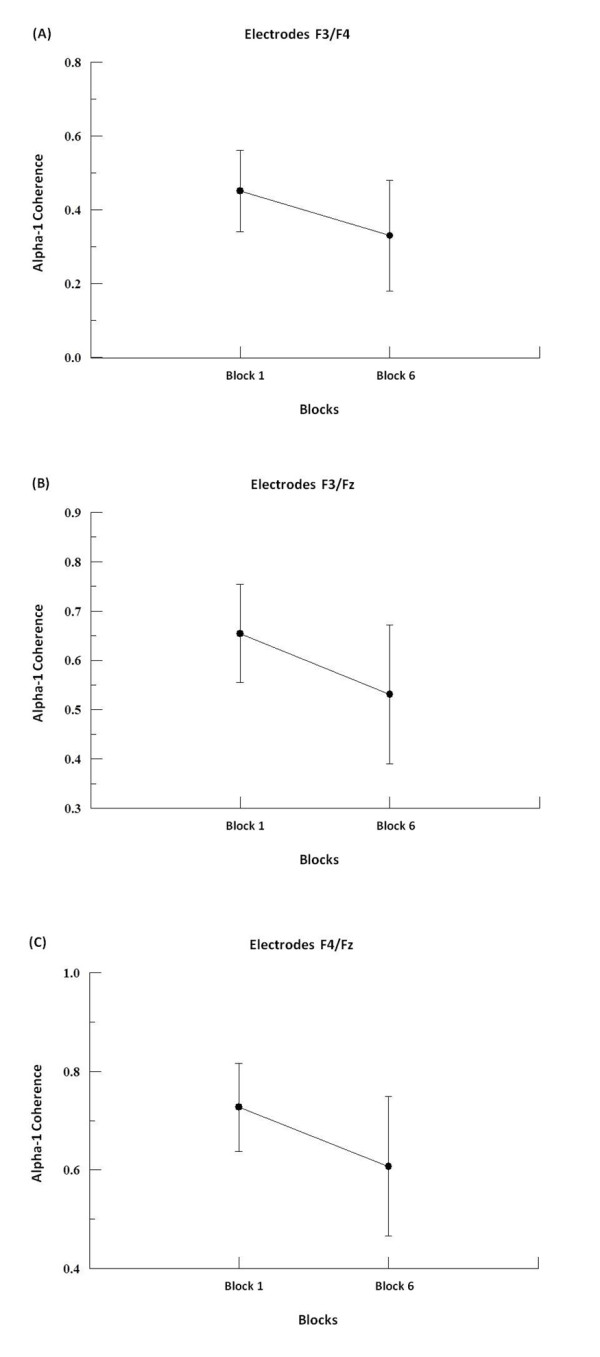
**Main effect for factor block observed through mean and SD**. (A) Comparison between block 1 and 6 in the electrodes F3-F4. Significant difference (p = 0.01). (B) Comparison between block 1 and 6 in the electrodes F3-Fz. Significant difference (p = 0.01). (C) Comparison between block 1 and 6 in the electrodes F4-Fz. Significant difference (p = 0.01).

**Figure 2:**
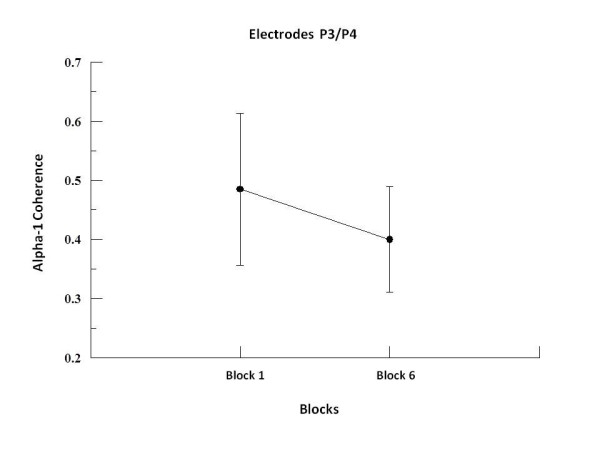
**Main effect for factor block observed through mean and SD**. Comparison between block 1 and 6 in the electrodes P3-P4. Significant difference (p = 0.01).

**Figure 3:**
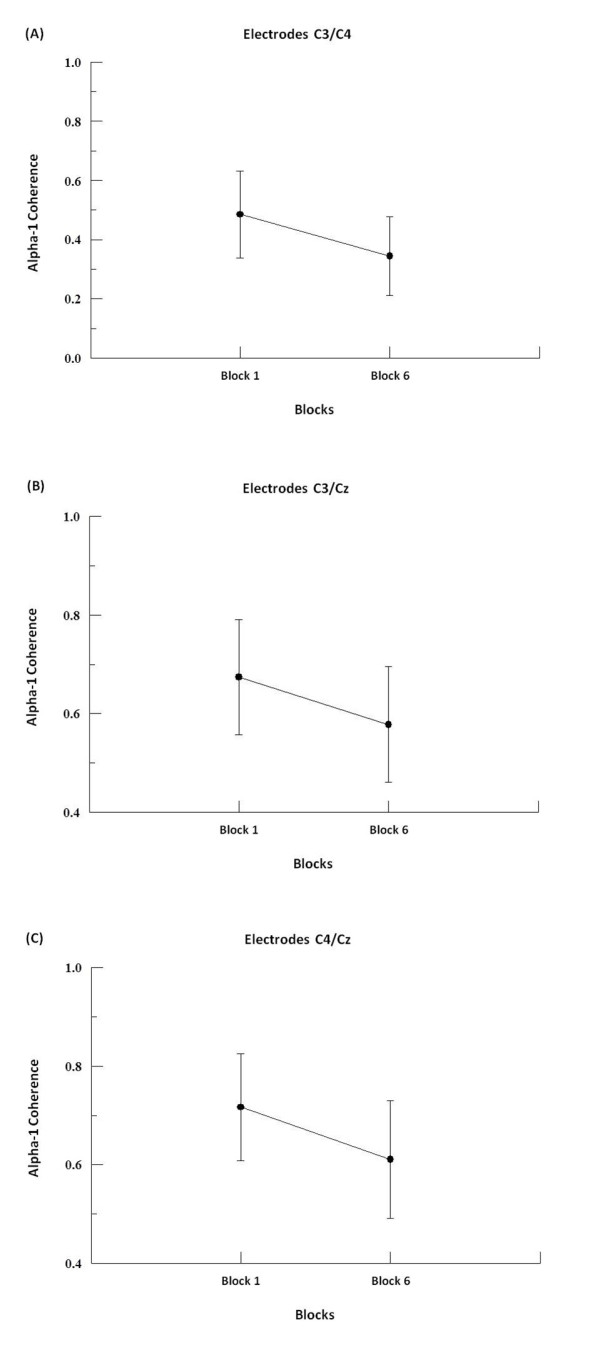
**Main effect for factor block observed through mean and SD**. (A) Comparison between block 1 and 6 in the electrodes C3-C4. Significant difference (p = 0.01). (B) Comparison between block 1 and 6 in the electrodes C3-Cz. Significant difference (p = 0.01). (C) Comparison between block 1 and 6 in the electrodes C4-Cz. Significant difference (p = 0.01).

**Figure 4:**
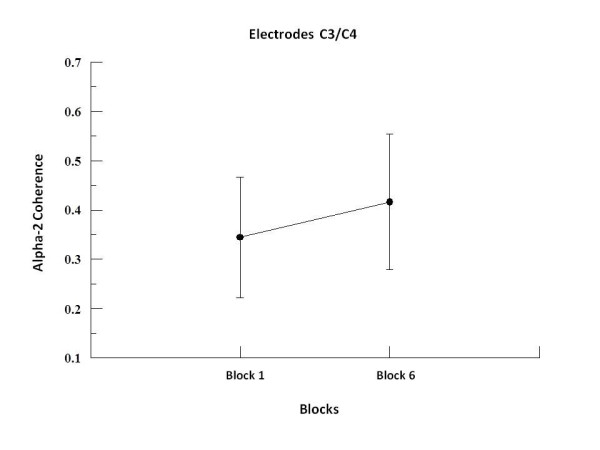
**Main effect for factor block observed through mean and SD**. Comparison between block 1 and 6 in the electrodes C3-C4. Significant difference (p = 0.01).

**Figure 5:**
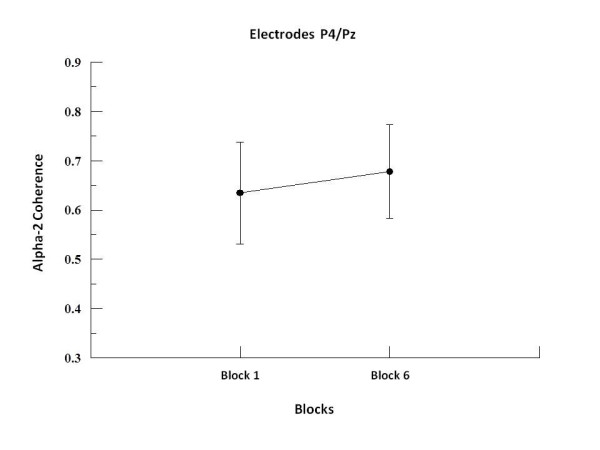
**Main effect for factor block observed through mean and SD**. Comparison between block 1 and 6 in the electrodes P4-Pz. Significant difference (p = 0.01).

**Figure 6:**
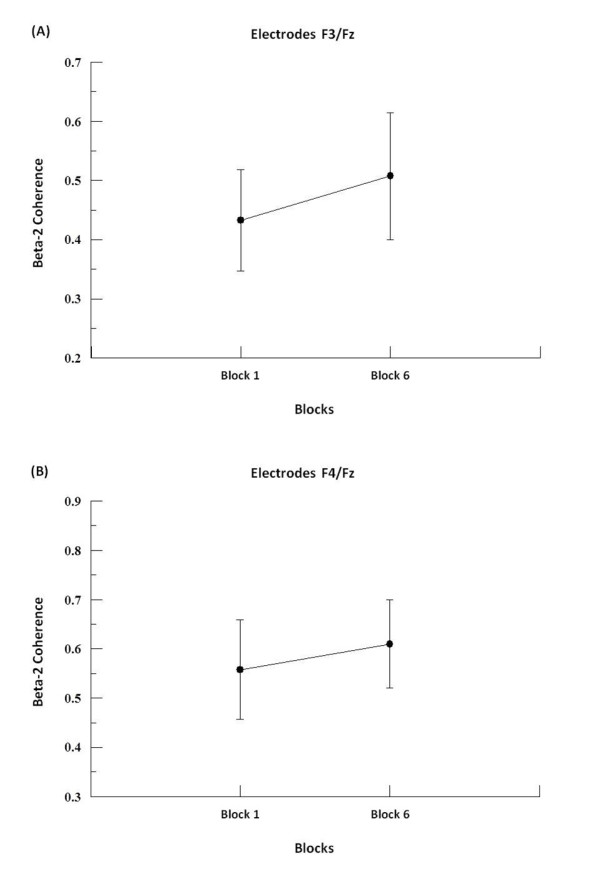
**Main effect for factor block observed through mean and SD**. (A) Comparison between block 1 and 6 in the electrodes F3-Fz. Significant difference (p = 0.01). (B) Comparison between block 1 and 6 in the electrodes F4-Fz. Significant difference (p = 0.01).

**Figure 7:**
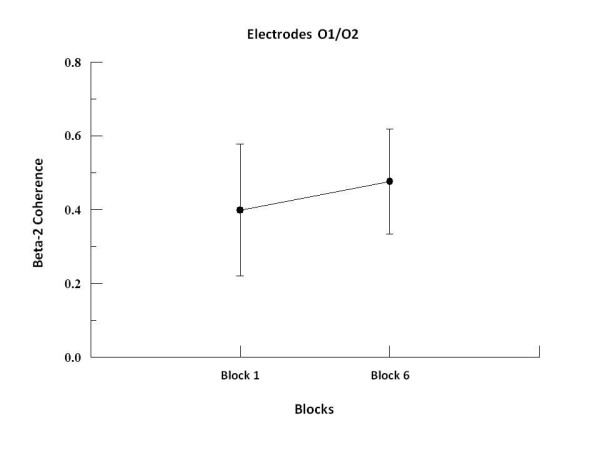
**Main effect for factor block observed through mean and SD**. Comparison between block 1 and 6 in the electrodes O1-O2. Significant difference (p = 0.01).

We found by two-way ANOVA main effects for factor block in alpha-1, alpha-2 and beta-2. In alpha-1, the two-way ANOVA revealed to F3-F4 (p = 0.001; F = 17.09), F3-FZ (p = 0.001; F = 20.18), F4-FZ (p = 0.001; F = 23.19) (see Figure 1), P3-P4 (p = 0.008; F = 7.50; see Figure 2), C3-C4 (p = 0.001; F = 19.99), C3-CZ (p = 0.001; F = 13.184) and C4-CZ (p = 0.001; F = 16.95; see Figure 3). In alpha 2, the two-way ANOVA revealed to C3-C4 (p = 0.017; F = 5.98; see Figure 4) and P4-PZ (p = 0.048; F = 3.67) (see Figure 5). In beta 2, the two-way ANOVA showed for F3-FZ (p = 0.001; F = 11.50), F4-FZ (p = 0.018; F = 5.80) (see Figure 6), O1-O2 (p = 0.036; F = 4.56) (see Figure 7). In addition, regarding the paired t-test, we found a decrease in coherence between the first and the last block (i.e., block 1 vs. 6) in all pairs of electrodes (p = 0.01).

## Discussion

This study aimed to bring new insights into the relationship between alpha and beta bands of qEEG within sensorimotor and associative areas involved in anticipatory actions, through coherence analysis, in a ball catching task. In particular, through the subdivision of alpha (i.e., alpha-1 and alpha-2) and beta (i.e., beta-1 and beta-2), we tried to elucidate electrocortical mechanisms within four target regions, i) frontal (F3-F4, F3-FZ, F4-FZ); ii) central (C3-C4, C3-CZ, C4-CZ); iii) parietal (P3-P4, P3-PZ, P4-PZ); iv) and occipital (O1-O2, O1-OZ, O2-OZ). We hypothesized that due to the task features, e.g. stimulus identification and catching of objects, we will find different patterns of activation during task execution.

### Frontal region

Our results have shown a decreased in alpha-1 coherence for the electrode pairs F3-F4, F3-FZ e F4-FZ between blocks in the ball catching task. The absence of a main effect for moment demonstrates that there was no difference between the moments before and after the balls drop. The coherence parameter was not sensitive enough to detect changes in the relationship between sub-areas of the frontal cortex. Factors such as expectation and attention prior to the fall of the object did not alter the functional connectivity between the three regions investigated on the scalp. In contrast, the block factor was altered in almost all the frequencies for all the electrodes sites analyzed. Essentially, the block factor was included in the analysis because we expected to see the effects of adaptation over the blocks, and other possible changes on cognitive functions, such as information coding (intrinsic aspects of the object, speed and oculo-manual coordination), motor-action planning, attentional processes and long-term memory. The electrode pair chosen represents the medial frontal cortex, which is related to the planning and programming of motor action [32].

In this context, our results showed a decreased in alpha-1 coherence between the first and the last block, suggesting that at the beginning of the task that corresponds to the first block, there is a need for a greater coupling between specific areas in the frontal region. The increased coupling in the frontal areas in the first block when compared to the last block, highlights different aspects of the involvement of the frontal cortex in the performance and adaptation to the processes of sensorimotor integration in the ball catching task. In this way, the decreasing coherence in alpha-1 between blocks may be seen as a consequence of the brain needs to adjust its responses required by the task, indicating, for example, that in voluntary and selective attention processes, the decreased coherence in alpha-1 occurs as an inhibitory response to disturbing cognitive processes [16,33]. Complementing the above findings, we also found a greater coherence in the right hemisphere compared to the left when observing the behavior of F3-FZ and F4-FZ electrodes in alpha-1, suggesting a greater specialization of the dominant hemisphere. This finding indicates greater automaticity of the right hemisphere when recruiting neuronal populations directly linked to the task specificity [1,11,33].

Our findings in beta-2 indicate an increased coherence for the electrode pairs F3-FZ e F4-FZ when comparing the first and the last blocks. In other words, we verified a greater communication between the left and the medial frontal cortex, as well as between the right and the medial frontal cortex, in the last block. The performed task required catching a free falling object, involving planning and selection of the motor response. As it is well known, the frontal cortex is directly involved with those processes [1,11]. Our results demonstrated that during the task execution (i.e., from the first to the sixth block) the functional connectivity between neuronal networks in frontal areas was reinforced. Other studies have demonstrated the relationship between beta-2 and the preparation [7] and execution of the motor act, that is, the beginning of the movement [27]. In this sense, our findings suggest that beta-2 is associated with the perception and spatial selection of response in accordance with the position and posture of the limb [6,34], with the establishment of new neural networks [34], and the trajectory of the limb [6]. These aspects suggest that the increased coherence in the frequency band beta-2, in medial frontal areas, would be responsible for coordinating new neural networks [7,35,36], and that the greater coherence found in the last block of the task could be viewed as a learning process [34,37,38].

### Central region

Our results in this region have shown decreased alpha-1 coherence for the electrode pairs C3-C4, C3 and C4-CZ-CZ between the first and sixth block of the task. These electrodes sites represent the somatomotor cortex, which is associated to sensory facilitation and preparatory behaviors (i.e. movement preparation) [3], motor execution [28], temporal organization and coordination of sequential movements [39], and also to the planning and organization of future actions [40]. Our data did not reveal any significant difference between C3-CZ and C4-CZ, even though these pairs of electrodes have shown a trend towards a decreased coherence for alpha-1band. In this context, our results suggest that the decreased in alpha-1 coherence (C3-C4), might be associated with preparatory attention to the stimulus identification, characterizing the sensory facilitation process [41] and the planning and preparation of motor gestures [3] that occurred during the blocks. Our findings also corroborate the hypothesis that learning of sequential actions is represented by pre-activation of the somatomotor region [39,42], which during the early stages of learning is associated with the prefrontal cortex to be involved in the decision and selection of movements, as well as in attentional processes [3,39,40]. Such findings also corroborate for the hypothesis that the premotor and primary motor cortices are activated in an integrated manner during the preparation of the motor act [43,44]. We also found an increased coherence in alpha-2, for the electrode pairs C3-C4, between the first and sixth block of the task. Such increase suggests a different role for alpha-2 in relation to learning processes [26,45]. Executive functions in central regions related to memory processes (working and long-term memory) are generally associated with theta oscillations [11,13], however, some studies have demonstrated an association between alpha-2 and long-term memory [26,45]. Hence, our results provide evidence that alpha-2 is related to processes of consolidation and retrieval long-term memory aspects, i.e., with the learning of the task and the recruitment of this information over the blocks. Other studies on unimanual tasks, showed an increase coherence (i.e., alpha band) (without regard to its sub-rhythms) in sensorimotor regions, along with the increased flow of information between brain regions, indicating that the relationship between alpha-2, working and long-term memory, are not yet conclusive [14,46].

### Parietal region

Our findings showed a decreased coherence in P3-P4 for alpha-1. The medial parietal cortex is associated with the integration of sensory information of different modalities and complexity, such as visual attention, anticipation, selection, and memory of visual stimuli [1,47]. Our result for alpha-1 in P3-P4 electrodes are probably related to the individual's performance in multisensory integration activities. In our study subjects integrate somatosensory/proprioceptive and visual stimuli according to the task demands, corroborating experiments have demonstrated to be possible to predict performance on a sensorimotor task through the coherence in alpha band [48]. Nevertheless, alpha-1 was also associated with the availability of global resources in the brain, including a greater attentional demand [49,50], and thus indicating that there was an optimization along the blocks to meet the integration demands of the task.

In contrast, our findings demonstrated an increased coherence in alpha-2 frequency band for the pair of electrodes P4-PZ. This intra-hemispheric increase may possible be related to the involvement of parietal cortex in sequential motor learning [51] in this case indicating an adaptation along the blocks for new learning acquisitions, and associating alpha-2 to long-term memory and to implicit attentional processes [52]. Another proposition is that a greater functional coupling of alpha in the parietal region occurs during the preparation of motor action [51]. Our findings are consistent with this hypothesis if we consider that the increased coherence in alpha-2 in the right hemisphere demonstrate the inhibition of the contralateral limb in response to new information acquisition, reflecting the role of this hemisphere on motor performance [2,14].

## Conclusion

In order to elucidate the cortical mechanisms involved in anticipatory actions, we suggested that different patterns of cortical activation could appear during the execution of the task. However, factors such as expectations and prior attention did not appear to be relevant enough to change the functional connections among the brain regions investigated. In this way, the factor block was added to our analysis, showing adaptation effects, or changes in all frequency bands for the electrode pairs analyzed. These adjustments are possibly related to the encoding of information (intrinsic aspects of the object, speed and eye-hand coordination), motor planning and attentional and long-term memory processes.

It was concluded that to perform tasks involving anticipatory actions, there is a great involvement of sensorimotor and associative areas, due to organization of information to manipulate the object, and to process visual-spatial information on the falling object and the hand contact. At last, we recommend further experiments using different objects, time randomization, samples with neurological and psychiatric disorders, and the observation of other neuro-behavioral variables.

## Competing interests

The authors declare that they have no competing interests.

## Authors' contributions

FS, SM, OAC, PR and AEN designed, conducted the literature review and drafted most of the manuscript. BV, FP, ST, MC, CP, LFHB, RP, MMG performed the literature review and the drafting of the manuscript. All authors were equally involved in reading and approving the final manuscript.
